# Acceptability and feasibility of peer-administered group interpersonal therapy for depression for people living with HIV/AIDS—a pilot study in Northwest Ethiopia

**DOI:** 10.1186/s40814-021-00889-x

**Published:** 2021-07-28

**Authors:** Biksegn Asrat, Crick Lund, Fentie Ambaw, Marguerite Schneider

**Affiliations:** 1 Alan J Flisher Centre for Public Mental Health, Department of Psychiatry and Mental Health, University of Cape Town, Cape Town, South Africa; 2 Centre for Global Mental Health, King’s Global Health Institute, Health Services and Population Research Department, Institute of Psychiatry, Psychology and Neuroscience, King’s College London, London, UK; 3 School of Public Health, College of Medicine and Health Sciences, Bahir Dar University, Bahir Dar, Ethiopia; 4 Department of Psychiatry, College of Health Sciences, Addis Ababa University, Addis Ababa, Ethiopia

**Keywords:** Acceptability, Feasibility, Group interpersonal therapy, HIV/AIDS, Ethiopia

## Abstract

**Background:**

Psychological treatments are widely tested and have been effective in treating depressive symptoms. However, implementation of psychological treatments in the real world and in diverse populations remains difficult due to several interacting barriers. In this study, we assessed the acceptability and feasibility of peer-administered group interpersonal therapy for depressive symptoms among people living with HIV/AIDS in Northwest Ethiopia.

**Method:**

We conducted a single-arm, peer-administered, group interpersonal therapy intervention with eight weekly sessions from 15 August to 15 December 2019 among people living with HIV/AIDS in Northwest Ethiopia. Four interpersonal therapy groups were formed for the intervention with a total of 31 participants.

**Results:**

Of the 31 recruited participants, 29 completed the intervention providing a retention rate of 93.5%. The process of the intervention and its outcomes were highly acceptable as most participants expressed success in resolving their psychosocial problems, adjusting to life changes and coping with stigma. The intervention was also reported to be feasible despite anticipated barriers such as access to transportation, perceived stigma and confidentiality concerns. The post-intervention assessment revealed significant reduction in depressive symptoms (mean difference (MD) = 9.92; *t* =  − 7.82; 95% CI, − 12.54, − 7.31; *p* < 0.001), improvement in perceived social support (MD = 0.79; *t* = 2.84; 95% CI, 0.22, 1.37; *p* = 0.009) and quality of life (MD = 0.39; *t* = 4.58; 95% CI, 0.21, 0.56; *p* < 0.001).

**Conclusion:**

Group interpersonal therapy is feasible and acceptable, and people living with HIV/AIDS can benefit from group interpersonal therapy in managing depressive symptoms and in improving perceived social support and quality of life. Future studies should examine the effectiveness of group interpersonal therapy in this setting.

**Supplementary Information:**

The online version contains supplementary material available at 10.1186/s40814-021-00889-x.

## Key messages regarding feasibility

What uncertainties existed regarding the feasibility?

- The relevance of interpersonal therapy (IPT) in the management of depressive disorders was well understood, but there was no established data on the feasibility of peer-administered group IPT for people with comorbid depression and HIV/AIDS in under resourced settings.

What are the key feasibility findings?

- Despite experienced barriers, peer-administer group IPT was considered acceptable to and feasible for people with comorbid depression and HIV/AIDS and for peer counsellors.

What are the implications of the feasibility findings for the design of the main study?

- This study found preliminary effectiveness in reducing depressive symptoms, improving social support and quality of life of people with depression and HIV/AIDS. The overall results of this study inform how to design, implement and evaluate future intervention trials in Ethiopia and in sub-Sahara Africa.

## Background

Psychological treatments have been widely tested and have shown effectiveness in treating depressive symptoms and other common mental disorders [1–4]. The use of evidence-based and culture-sensitive psychological interventions has attracted the scientific community as a result of their promising effectiveness and replicability even in low-income settings [1]. Hence, the World Health Organization (WHO) advocates access to evidence-based psychological treatments for moderate to severe depressive disorders after following proper adaptation procedures [5]. However, implementation of psychological treatments in diverse populations and in different settings remains difficult due to several interacting barriers during the implementation process [1, 6] in a manner that may not always be evident when implementing physical health interventions. Implementation is often undermined by problems related to low acceptability and feasibility of the psychological interventions in a new context [6, 7]. Several factors contribute to low acceptability and feasibility, such as lack of resources and access, workload on task-sharing interventionists, low retention and inadequate level of competency of interventionists [7].

In LMICs, common barriers for implementation of evidence-based psychological treatments include (i) lack of cultural adaptation of psychological treatments in new populations [8, 9], (ii) lack of evidence on the applicability of new interventions despite anticipated barriers [10, 11] and (iii) low mental health literacy of the community limiting their uptake of an intervention [12].

Psychological treatments may not always need to involve highly trained health professionals [6, 13]. They can be delivered by peer counsellors effectively if appropriate training and supervision are provided [1]. However, it is important to establish whether the treatment is acceptable and feasible for a new population and in a new setting. Therefore, proper adaptation and systematic harmonization are fundamental to improve ecological validity (the degree of congruence between the intervention and the environment), cultural sensitivity and effectiveness of interventions for new populations [14, 15]. Moreover, examining the acceptability and feasibility of psychological treatments is crucial before moving into full-scale evaluation of effectiveness.

Acceptability is the perception of those receiving the treatment, or any other stakeholders, on whether a given intervention and its outcomes are applicable and satisfactory [1, 6, 16]. From a research perspective, it allows us to determine whether an adequate number of subjects can be recruited for a larger study [16, 17]. Feasibility is the extent to which an intervention can be provided successfully for a specific population despite constraints [16]. It examines overall barriers and facilitators to the implementation of an intervention such as applicability (deliverable as planned) and practicality (delivered despite barriers) [18, 19]. Feasibility evaluation is particularly important for the implementation of psychological treatments in low-resourced settings and in communities with different cultural backgrounds where the context of implementation may differ from the one where it was originally developed [18].

Interpersonal therapy (IPT), a well-tested psychological treatment in high-income countries, has shown efficacy for the treatment of depressive symptoms in a variety of populations [13]. A recent systematic review and meta-analysis found that group IPT was effective in reducing depressive symptoms in people living with HIV/AIDS (PLWHA) in LMICs [20] and that most of the triggering factors for depressive symptoms were interpersonal problems originating from the problem areas of IPT. The four problem areas of IPT are (i) disagreement or conflict, (ii) life change (negatively or positively), (iii) loneliness or social isolation and (iv) grief [21].

IPT allows for the exploration of clients’ interpersonal problems, finding the links between interpersonal problems and onset of depressive symptoms and making adjustments to alleviate depressive symptoms [13, 22]. The group format of IPT is believed to increase coverage and reduce costs of the intervention [13]. Furthermore, it helps counsellors to identify and work on specific problematic communication patterns during interactions among group participants to address interpersonal problems [13].

Psychological treatments that can be administered by lay counsellors including by peers are highly relevant to overcome the lack of trained human resources in low-income settings. Peer-administered counselling and education interventions have shown promising results in reducing depression and in improving overall mental health outcomes for adolescents in LMICs [23]. Peers are knowledgeable on the context and can speak the local language. However, their acceptability by the community they are serving and their competency to deliver the intervention successfully needs to be addressed [24]. Importantly, the selection, training and supervision of peers are fundamental procedures that should be clarified when considering implementing peer-administered psychological treatments.

In this study, we aimed to assess the acceptability and feasibility of peer-administered group IPT for the treatment of depressive symptoms among a selected group of people living with HIV/AIDS (PLWHA) in Northwest Ethiopia. We also examined the retention rate and the effect of the intervention in reducing depressive symptoms and improving quality of life (QoL), functionality and social support by comparing baseline and post-intervention assessments. Although peer-administered psychological treatments are widely implemented in a task-shifted approach in LMICs [25–27], the acceptability and feasibility of peer-administered group IPT have not been explored in Ethiopia. The current study was the first effort to pilot group IPT in an HIV population in Ethiopia.

## Methods

### Study design and setting

A single-arm intervention study coupled with pre- and post-assessments was used from 15 August to 15 December 2019 among PLWHA who attended antiretroviral therapy (ART) follow-up appointments during the study period at Felege-Hiwot Referral Hospital (FHRH) in Northwest Ethiopia. We used peer-administered group IPT intervention with eight weekly sessions for treatment of depressive symptoms and examined the acceptability and feasibility of the group IPT for PLWHA in Ethiopia. A detailed description of the setting is available elsewhere [21]. This paper reports on a feasibility and acceptability pilot study. The CONSORT 2010 to the pilot and feasibility checklist [28] was used to report the findings of this study. Items that were not applicable to this study, such as randomization and blinding items were omitted as there was no control group.

### Procedures

#### Selection of trainers, supervisors and potential counsellors

Group IPT trainers were selected from the academic staff in the Department of Psychiatry in Bahir Dar University. The selected group IPT trainers were mental health professionals with a Master’s level educational background who studied clinical and community mental health for at least 2 years. They had completed the group IPT training of trainers’ course and had 5 to 10 years’ experience in organizing and facilitating mental health training for health professionals. In addition to their academic work, they were also providing clinical services at the Psychiatric Clinic in FHRH. They provided the group IPT training for supervisors and potential counsellors in accordance with the adapted WHO group IPT manual [29].

In consultation with stakeholders of the ART clinic, we set the minimum criteria for selecting potential counsellors. These were a minimum of secondary education (grade 10 and above), being an adherence supporter, case manager, HIV educator or community health worker at the ART Clinic, involved in the direct care of PLWHA in the last one year, and being highly motivated to help others. After further discussions with the trainers and supervisors, we decided that the most suitable people to do the counselling were the adherence supporters and case managers. Case managers and adherence supporters were most suitable to provide the intervention because of their shared lived experiences with difficult life circumstances, views about cause and treatment of depression, and to minimize stigma and maintain confidentiality [21]. In our prior study results, we found that case managers and adherence supporters viewed psychosocial problems as the main cause of depression. They also believed that depression can be treated through psychosocial support and counselling [21].

#### Group IPT training for candidate counsellors and supervisors

A total of 12 people (ten candidate counsellors and two candidate supervisors) received intensive group IPT training for 7 days guided by the WHO group IPT curriculum [29]. The duration of the training was decided in consultation with mental health professionals and based on group IPT trainers’ recommendations. The training was facilitated by four group IPT trainers—two trainers led the morning sessions and the other two facilitated the afternoon sessions. The training included brainstorming, brief presentations, group discussion and predominantly role plays to demonstrate group facilitation skills. Daily training evaluations were conducted at the end of each training day and the trainers adjusted the training to accommodate given comments. At the completion of the training, all trainees took an exit test to assess their level of competency. The test was developed by the WHO group IPT team that included 15-item questions available at the appendix of the manual [29]. The score was calculated based on the scoring instruction indicated in the manual as follows: 2 points if the question was answered adequately, 1 point if important information was missing, and 0 if it was not answered correctly. The WHO-IPT guideline recommends that group IPT trainees should achieve a score of at least 70% to effectively facilitate the group IPT intervention [29]. Of the ten candidate counsellors, six of them scored above 70% and we recruited them to facilitate the group IPT intervention as counsellor. The counsellors were named “peer counsellors,” because (i) both adherence supporters and case managers are HIV positive, (ii) all of them reported having experienced depressive symptoms at some point in their lives, similar to the participants. The main responsibilities of counsellors are indicated in Table 1.

**Table 1:** The main responsibilities of peer counsellors

• Receiving eligible participants, getting consent, conducting pre-group counselling and documenting participants’ physical address and telephone number
• Forming IPT groups, setting a meeting date, time and place
• Evaluating depressive symptoms of each participant, taking attendance notes
• Facilitating the group counselling (conducting problem inventory, encouraging participants to generate possible solutions, make decision analysis, set strategies, providing guidance and counselling)
• Referring clients who refused to participate in the group IPT and transferring severely sick or dangerous clients to the psychiatric clinic

The two nurse professionals (HT and TG) were also found competent in mastering the group IPT (scored above 70% on the exit test). The group IPT trainers provided brief supervision training for HT and TG for one additional day after the completion of the group IPT training. This focused on how to conduct constructive and effective supervision specifically, when and how to conduct supervision, how to conduct fidelity assessment for each group session, and when and how to exchange feedback with counsellors and the PI. Both HT and TG practiced supervision in role plays in front of the trainers before being recruited as supervisors. Each supervisor practiced real supervision in three IPT sessions under supervision of the trainers before conducting supervision independently as recommended in the manual [29].

The main responsibilities of the supervisors were supporting and organizing the overall delivery of the project and maintaining contact with stakeholders such as the PI, counsellors and staff members working at the ART clinic. This includes overseeing, monitoring and evaluating the progress of the intervention, coordinating the intervention team and checking fidelity. Supervisors observed each IPT group during sessions at least every 2 weeks. They also listened to the audio-recorded sessions and evaluated fidelity of each session. They were also responsible for evaluating group sessions using fidelity checklists, to provide feedback to counsellors and to report to the principal investigator.

#### Theory of change workshop

A Theory of Change workshop was conducted to identify available resources, potential barriers and methods to implement the intervention 1 month before the initiation of the intervention. A theory of change can be defined as “a particular approach for making underlying assumptions in a change project explicit, and using the desired outcomes of the project as a mechanism to guide project planning, implementation, and evaluation” [30]. The theory of change workshop was used as a tool to bring everyone on board in the planning and to tailor the intervention implementation to fit with the context of the implementation. Case managers, adherence supporters, mental health professionals, group IPT trainers, nurses and a unit leader at the ART clinic participated in the workshop. Workshop participants explored problems that affect the life of PLWHA, intervention strategies for group IPT, and set assumptions and indicators. The assumption that *managing depression improves social support, functioning and quality of life* was identified as a prior aim to the intervention. Moreover, the availability of meeting spaces, human resources available to be trained as counsellors, and staff good will and motivation as facilitators for implementation of the group IPT. Limited access to transportation, fear of stigma, poor help-seeking behaviour, low understanding of the public about the disabling nature of depression, and illiteracy of most PLWHA (for documenting homework during the intervention) were identified as barriers to the implementation of the intervention (Supplementary file 1).

### Recruitment of participants for the intervention

Participants of the intervention were recruited from a sample enrolled in a randomly selected cross-sectional survey of PLWHA (*N* = 393) attending the facility from 8 July to 7 October 2019. Results of the survey are published elsewhere [31]. A formal sample size calculation may not be appropriate for pilot studies [32]; therefore, participants were recruited into the intervention consecutively from the survey [31] until five to ten participants were recruited every week to form IPT groups. The number of participants in each IPT group was decided to be between five and ten based on previous study findings [21], and the maximum waiting time for eligible participants before starting group sessions was determined to be 1 week. The findings of the survey indicated a high prevalence of major depressive disorder (32.5%). Major depressive disorder was positively associated with reduced adherence to ART treatment and negatively associated with quality of life. When participants were found eligible for the intervention during the survey [31], they were consented and immediately referred to the group IPT intervention. The survey assessment served as a baseline for the pilot study.

Participants who (i) were adults aged 18 years old or above attending clinical follow-up at the ART clinic of FHRH, (ii) scored five or more on a locally validated Amharic version of the Patient Health Questionnaire (PHQ-9), and/or (iii) were diagnosed with major depressive disorder (MDD) using the Mini International Neuropsychiatric Interview (MINI) were referred by research assistants to group IPT counsellors immediately. Counsellors checked clients’ documents for eligibility and approached them to invite them to participate in the intervention. Exclusion criteria for the intervention were (i) being severely sick with a major physical illness or needing urgent medical attention (for example, TB patients), (ii) those identified as being a danger to themselves or others, such as suicidal or homicidal ideation, (iii) having emotional problems as a result of an organic cause or substance use—detected by research assistants using the MINI exclusion criteria for MDD, (iv) having a major depressive disorder with psychotic or catatonic features and (v) those who were diagnosed with cognitive deficits or Tuberculosis by ART clinicians or as indicated in the patient’s clinical chart. Group IPT counsellors provided a detailed explanation on the purpose and content of the intervention.

**Fig. 1:**
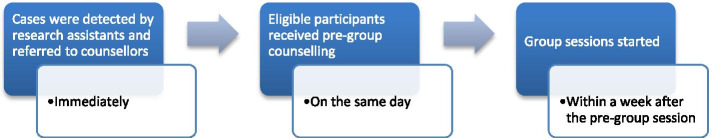
Procedures of recruitment into the pilot study

Baseline information of each participant who agreed to join the intervention was then documented into a patient folder. IPT groups were formed as soon as six to ten participants agreed and consented to join the intervention. Group sessions were started within a week after groups were formed. Session days in the week and regular meeting times were set according to the availability of all participants in that group (Fig. 1).

At the end of the intervention, we randomly selected a total of 12 group IPT participants (three participants from each IPT group), for in-depth interviews to explore their views on the acceptability and feasibility of group IPT for PLWHA in Ethiopia. We also recruited all of the counsellors (*n* = 6) for a focus group discussion to elicit their opinions on the acceptability and feasibility of the group IPT intervention. Four of the counsellors were case managers and two of them were adherence supporters who were supporting staff members at the ART clinic. Both case managers and adherence supporters are living with HIV/AIDS and had similar lived experience as most of the PLWHA. The participants received group IPT training for 7 days and were found to be competent to deliver the intervention based on the group IPT competency assessment.

### Implementation

The intervention was guided by the adapted WHO group IPT manual [21]. Prior initiating the intervention, we completed a substantial process of adapting the original WHO group IPT manual into the local context. The manual adaptation included language and content adaptations. First, we conducted language adaptation for the original manual using a stepwise approach to make a language-appropriate intervention. We included local names, terminologies, slang terms, symbols and quotes to make the language used in the manual understandable to lay people. For content adaptation, we explored problem areas for intervention and strategies for intervention implementation. We followed several steps while conducting the adaptation using the *Participatory and Iterative Process Framework for Language Adaptation* (*PIPFLA*) [33]. Initially, the original manual was translated into Amharic by HT then back translated by TG. The harmonized document and the original manual were sent to four scientific reviewers (three of them were mental health professionals and one of them was a clinical psychologist) for review and their comments incorporated by BA. Finally, a half day adaptation workshop was conducted to further revise the manual. This helped to determine preferred counsellors, group size and dynamics, meeting space and place, and suggested number, duration and frequency of sessions. Several stakeholders participated in the workshop such as nurses working at the ART clinic, adherence supporters, case managers, head of the ART clinic and mental health professionals. The final harmonized manual was used in the training for this pilot evaluation.

The intervention comprised of eight weekly 90-min sessions structured into three phases: *initial*, *middle and termination phases*. Two counsellors were assigned per IPT group (one mainly documenting and the other leading the discussion, but neither limited to these activities). The sessions were conducted at the ART clinic in rooms arranged to accommodate 10 to 12 people at a time.

Prior to joining group sessions, every participant received pre-group counselling (one individual session). During the pre-group session, counsellors assisted each participant to recognize depressive symptoms, to link their interpersonal problems with depressive symptoms and to set strategies and goals for solving the problems. The *initial group phase* (session 1) included a brief discussion on depression and its impacts, and the IPT problem areas (intervention components), followed by presentation of their own depressive symptoms by each participant and exploration of the links between depressive symptoms and the problem areas. In this initial group session, the group members agreed on group goals that should be achieved in the next seven sessions. In the *middle phase* (sessions 2 to 7), group participants developed strategies to address the identified problems and to implement selected strategies. Depressive symptoms and the links to problem areas were reviewed every week from sessions 2 to 7. The strategies were evaluated in every session and participants shared stories related to their success to encourage others. Homework and practical exercises were given every week. Homework was given based on identified problem areas that contributed for their depressive symptoms. For example, if someone’s problem was a dispute with one of the family members (with their wife or husband) at home, s/he was given homework to start having a calm conversation with him/her. In the *termination phase* (session 8), depressive symptoms and problem areas were reviewed and plans set for each participant. Discussions were held on how to recognize a relapse in depressive symptoms and what they should do when they have symptoms. The implementation phases and respective activities are described in Table 2.

**Table 2:** The structure of the group IPT intervention implemented at the ART clinic in Felege-Hiwot Referral Hospital in Northwest Ethiopia

Phases	Sessions	Activities
Pre-group phase	Individual session	• Conducting symptom inventory guided by PHQ-9 items • Exploring links between onset of symptoms and interpersonal problems • Providing hope and help clients to join the IPT group
Initial phase	Session 1	• Facilitate the group members to introduce themselves to each other • General discussion about depressive symptoms and interpersonal problems • Facilitate participants to talk about their symptoms and triggering factors • Set group goals, strategies to solve problems and finalize the session.
Middle phase	Sessions 2–7	• Review symptoms of each participant • Link depressive symptoms to events from the previous week • Encourage participants to contribute problem-solving options • Decision analysis and implement the strategies to achieve the goals • Providing homework for every participant
Termination phase	Session 8	• Evaluate the number and severity of symptoms • Make plans, consider referral or consultation if symptoms persist • Discuss relapse of symptoms and actions to be taken

#### Review meetings

The implementation team (counsellors, supervisors and the principal investigator) met every week for an hour. The aim of the review meeting was to exchange feedback, evaluate the progress of the intervention and address challenges that affected the intervention. The meetings focused on improving the intervention and evaluating decisions made by the counsellors and supervisors.

#### Fidelity of the intervention

All group IPT sessions were audio-recorded for the purpose of fidelity evaluation. At completion of each session, fidelity was rated by the two supervisors (HT and TG) and the principal investigator (BA) independently using the WHO group IPT fidelity checklist which is available at the appendix of the manual [29]. Subsequently, identified gaps in adhering to the intervention protocol were communicated to the counsellors in a brief post-session meeting. Discrepancies in the scoring of fidelity items were resolved through discussion with the raters and the PI researcher (BA).

### Implementation outcomes

#### Acceptability

Participants were asked to provide their overall views and specific issues on the acceptability of the group IPT intervention. They were asked to provide their views on the number of sessions, session duration, frequency of sessions, competency of counsellors, group size and intervention site. They were also asked to describe their level of satisfaction with the sessions, process of the intervention, competency and approach of counsellors, the convenience and comfort of intervention location, handling clients’ information and confidentiality, and with the outcome of the intervention. They were also asked to rate their level of satisfaction with the overall process of the intervention with a range from very dissatisfied to very satisfied. At the completion of the intervention, counsellors shared their views on the acceptability of the intervention in the interviews and provided detailed suggestions on an acceptable approach for future use referring to their observations and experience of providing the intervention.

#### Feasibility

Feasibility of the intervention was explored using semi-structured open-ended questions. The questions were framed to explore the following concepts: (i) available resources and trained people to carry out the intervention, (ii) applicability of the intervention to the larger HIV population and (iii) compatibility of the intervention with existing health service systems. In addition, we calculated the retention rate by dividing the number of participants who completed the intervention by the number of participants who began the intervention to evaluate whether group participants were able to complete the intervention despite anticipated barriers.

#### Depressive symptoms, QoL, functioning and perceived social support

Participants who completed the intervention were reassessed for depressive symptoms, perceived social support, functioning and QoL using PHQ-9, Perceived Social Support (PSS-12), WHO Disability Assessment Schedule (WHODAS-12) and the adapted and brief version of the WHO Quality of Life assessment for PLWHA (WHOQOL-HIV-BREF-Eth) instruments respectively. A detailed description of these instruments is available elsewhere [31].

### Data collection

Qualitative data collection and pre-post assessments were conducted by independent research assistants who were trained for data collection purpose only. All of the research assistants were nurse professionals who had prior experience in mental health research with PLWHA and had received mhGAP training (a mental health training program designed to address mental, neurological and substance use disorders). We provided them with a 3-day practical training on qualitative and quantitative data collection applicable to the intervention. Qualitative data were collected using audio-recorded in-depth interviews and a focus group discussion from the group IPT participants and counsellors respectively. In-depth interviews were conducted with selected participants from each IPT group at the completion of the intervention. The in-depth interviews and the focus group discussion aimed at assessing acceptability and feasibility of the group IPT. The interview guide is available as supplementary file (Supplementary file 2).

At the completion of the intervention, the same measures used in the baseline were administered to all group participants to collect to evaluate the status of depressive symptoms, perceived social support, functioning and QoL. The post-intervention quantitative data were collected by one research assistant who did not participate in any way in the intervention.

### Analysis

A framework analysis was used to analyse the qualitative data and to explore the acceptability and feasibility of the intervention. The audio-recorded data were transcribed verbatim in Amharic and translated into English. The translated transcripts were double checked for accuracy and imported into NVivo 12 computer program for analysis. The data were analysed using predefined themes using a framework approach [34, 35]. The themes for acceptability were overall acceptability of the intervention, benefits gained from the intervention, satisfaction with counsellors, acceptability of the structure and process of the intervention. The themes for feasibility were general views on the feasibility of using the intervention for a larger population, barriers to the intervention and strategies to solve the barriers, feasibility of using peer counsellors, and general recommendations. The themes for acceptability and feasibility were selected from core elements of intervention adaptation and implementation science [1, 16, 17] and based on the scope of our study.

Quantitative data were analysed using SPSS version 26. We used descriptive statistics to summarize participants’ characteristics and to calculate mean scores. Paired samples *t* test analysis was used to compare the scores of depressive symptoms, QoL, functioning and perceived social support before and after the intervention, and to examine the direction of the change of the intervention. The paired-samples *t* test analysis was conducted after homogeneity and normal distribution assumptions were fitted in the model.

## Results

### Participant recruitment and retention

**Fig. 2:**
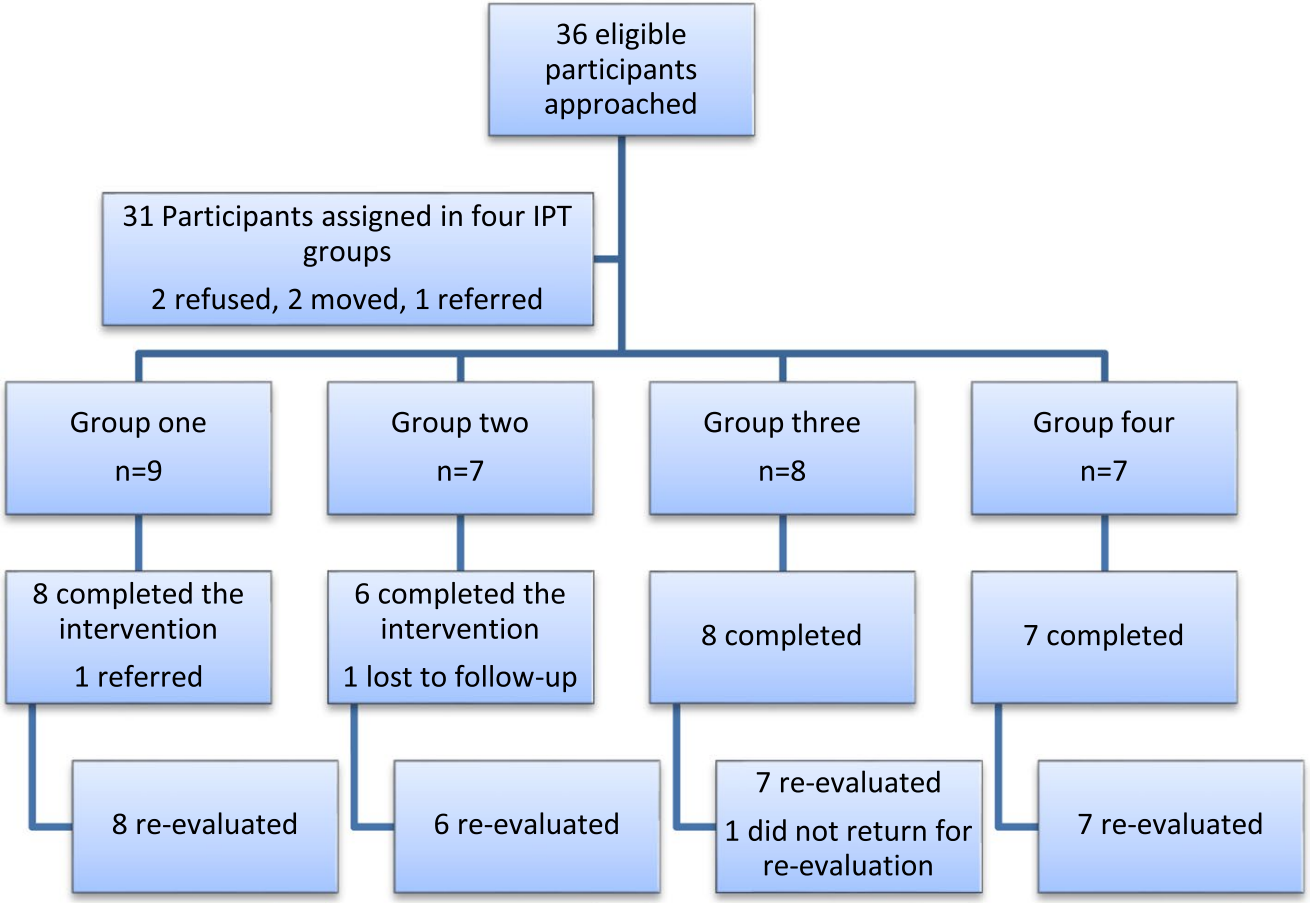
Recruitment, group formation and retention of group IPT participants

A total of 36 potential participants were approached and 31 participants were recruited and assigned into four groups for the intervention within a 1 month recruitment period. Of the total participants recruited for the intervention, 29 of them completed the intervention providing a retention rate of 93.5%. Among those who completed the intervention, 28 of them were reassessed for depressive symptoms, QoL, functionality and perceived social support 1 week after the end of the intervention and one participant did not return for re-assessment (Fig. 2). One of the participants from group 1, who had a previous history of psychosis, was referred to the psychiatric clinic as she started showing inappropriate behaviour and suspiciousness toward other group members at the 4th group session. One more participant from group 2 was lost after the fifth group session and the intervention team could not find him using his contact address. One of the participants from group 3 who completed the intervention refused to come for re-assessment.

### Sociodemographic characteristics of the participants included in the intervention

Of the total recruited participants for the intervention (*N* = 31), three quarters (*n* = 23, 74.2%) of them were women and 41.9% (*n* = 13) were either divorced or widowed. More than half of the recruited participants (*n* = 17, 54.8%) were illiterate and 38.7% (*n* = 12) were unemployed (Table 3).

**Table 3:** Baseline sociodemographic and clinical characteristics of the group IPT participants reassessed at the end of the intervention, Felege-Hiwot Referral Hospital, Northwest Ethiopia (*N* = 31)

Sociodemographic and clinical characteristics		*N* (%)
Age in years	18–29	6 (19.4)
	30–39	13 (41.9)
	40–49	9 (29.0)
	50 +	3 (9.7)
Gender	Male	8 (25.8)
	Female	23 (74.2)
Marital status	Single	7 (22.6)
	Married/in relationship	11 (35.5)
	Divorced/widowed	13 (41.9)
Educational status	Illiterate	17 (54.8)
	Completed primary education	9 (29.0)
	Completed secondary education	4 (12.9)
	Completed tertiary education	1 (3.2)
Employment	Public servant	2 (6.5)
	Self-employed	17 (54.8)
	unemployed	12 (38.7)
Current CD4 count	< 200	5 (17.9)
	200–349	4 (14.3)
	350–499	5 (17.9)
	> 500	14 (50.0)
ART treatment regimen	First-line treatment	17 (56.7)
	Second-line treatment	13 (43.3)
Duration of ART treatment in years	1–5	8 (26.7)
	6–10	11 (36.7)
	11 and above	11 (36.7)

Current CD4 count refers to the recent CD4 count within the last 6 months; *IPT* interpersonal therapy

### Sociodemographic characteristics of the in-depth interview and focus group participants

Of the total participants in the intervention (*N* = 12), nine of them were female and half (*n* = 6) of them were divorced. The median age was 37.5 and mean age was 38.8. Five of the in-depth interview participants were illiterate and six of them were unemployed. See Tables 4 and 5.

**Table 4:** Sociodemographic characteristics of in-depth interview participants

IPT group	Code	Age	Gender	Marital status	Educational status	Employment
Group 1	G1N1	50	F	Married	Illiterate	Unemployed
	G1N2	32	F	Married	6th grade	Unemployed
	G1N3	37	F	Divorced	6th grade	Self-employed
Group 2	G2N4	28	M	Married	Illiterate	Farmer
	G2N5	42	F	Divorced	Illiterate	Unemployed
	G2N6	65	F	Divorced	Illiterate	Unemployed
Group 3	G3N7	28	F	Married	12 + 3	Nurse
	G3N8	44	M	Married	8th grade	Unemployed
	G3N9	38	F	Divorced	10th grade	Unemployed
Group 4	G4N10	26	M	Single	10th grade	Wood worker
	G4N11	40	F	Divorced	Illiterate	Self-employed
	G4N12	35	F	Divorced	10th grade	Self-employed

**Table 5:** Sociodemographic characteristics of focus group participants (group IPT counsellors)

Code	Gender	Age	Educational status	Role at the clinic	Years on ART
C1	M	40	10 + 2	Case manager	6
C2	F	40	10th grade	Case manager	8
C3	F	35	11th grade	Case manager	12
C4	F	45	10 + 3	Case manager	9
C5	M	58	11th grade	Adherence supporter	8
C6	F	21	10 + 2	Adherence supporter	2

#### Acceptability

##### Overall acceptability

All the procedures used during the intervention and its outcomes were reported to be acceptable. Participants reported that they enjoyed the intervention and believed the existing culture supports group discussion.

Group 4, participant number 12 (G4N12): “I really enjoyed the group counselling …. the group was great in generating solutions for problems common to everyone such as disagreement and self-isolation from social participation. That is why I am coming every week to attend sessions.” (Female participant).

One of the participants considering “becoming a counsellor” after seeing how much group based counselling works in the community.

Group 3, participant number 7 (G3N7): “…. I have a diploma in nursing, and I have discussed with my husband about becoming a counsellor. I am confident that I can facilitate group sessions effectively if I can get the counsellors training.” (Female participant).

Counsellors also reported that the group IPT was acceptable because it was a medium that encouraged most of the participants in bringing change into their lives.

C4: “The group counselling was source of motivation for all of the participants. I have been observing a great change in their behaviour and facial expression. They appeared comfortable from session to session – they had warm greetings, free communications, and socialization with their friends.” (Female counsellor).

##### Perceived benefits

Qualitative explorations found that the intervention was useful to participants in several ways. The most common benefits reported were resolving social conflicts, gaining confidence to disclose their HIV status to their family members, and reducing depressive symptoms.

G1N1: “I had long lasting conflict with one of my neighbours. She (the neighbour) was a pain for me for a long time. Last week, I greeted her, and she hugged me with a smile. I felt as if a big burden has removed from my shoulder.” (Female participant).

Another participant stated the benefit she gained was to be able to disclose her HIV status: “After I attended the first few sessions, I have decided to disclose my HIV status to my closest people.” (Female participant).

##### Satisfaction with peer counsellors

Participants stated that they were happy with the peer counsellors and perceived them capable in facilitating group sessions. They said that the peer counsellors could understand common problems of PLWHA.

G2N6: “I am happy with the counsellors because all of them were our peers who passed through the same difficult life circumstances. They have given us a lot of hope and we feel we can be healthy as they are.” (Female participant).

Another participant said that the words of counsellors were encouraging and brought hope in everyone’s life.

G4N12: “They are very supportive like a family – like a mother and a father. … Their word is encouraging and gave us a lot of hope.” (Female participant).

However, one of the participants was not happy with the unprofessional behaviour of one of the counsellors because “she was dreading her hair” during the group discussion (Female participant).

##### Satisfaction with the intervention setting

Participants were happy with the meeting place and space. They said that attending group sessions in a more private place that has minimal social interruptions can facilitate open discussion. One participants said that group sessions can be conducted anywhere but feels uncomfortable to come to the ART clinics due to his “*bad memories*” at the ART. (Female participant).

##### Views on number and duration of sessions

Conducting one session weekly for a duration of 8 weeks was accepted by participants. However, some of the counsellors argued that 90 min is too short, while most counsellors argued that it was enough to review each group participant’s issues.

C4: “The 90 min session duration was short for me to cover all ideas coming from every participant. Everyone wants to talk from the heart…they want to talk more. Therefore, the time was short for me.” (Female counsellor). Counsellors were optimistic about the intervention bringing significant change for all group participants. They wanted to see every group participant treated for depression “until everyone feels healthy” and they suggested inclusion of more sessions “up to 10 or 12.”

#### Feasibility

##### General views on feasibility of the intervention and use of trained peer counsellors

IPT in a group format was perceived to be feasible by participants, as long as it is conducted in an accessible and private setting and an adequate number of peer counsellors are available. But few participants mentioned that the lack of trained peer counsellors could be a barrier to expanding the intervention. Counsellors believe that the group IPT can be feasible “as long as clients are responsive” (Female counsellor).

##### Experienced barriers

Participants experienced several barriers to attending group sessions such as access to transportation, employment, social responsibilities, sickness and stigma.

G1N1: “Initially, I thought that I had no energy to come every week because my home was far from the town. Later, I found it a good opportunity to go out of home for a good reason.” (Female).

G2N6: “Unfortunately, I did not come for two session due to family issues. My daughter gave birth and I was with her to take care of her. I could not come because of social responsibility towards my daughter.” (Female).

To most of the counsellors, the intervention was completed smoothly; however, some of them reported that phone calls were causing interruptions during sessions.

C5: “Initially, some clients were not silencing their phone calls but later everyone silenced their phone after we included it in the list of the group rules.” (Male counsellor).

In addition, presentation of new and bigger problems in every session by participants was perceived as a challenge for the intervention to achieve the initial goals.

C3: “Some problems presented by participants were not achievable in the short term. When we focused on achievable problems, they became less interested and they took the group discussion into the biggest problem that we could not fix it alone.” (Female counsellor).

##### General recommendations received

Group participants recognized the burden of depression in people with HIV/AIDS and they recommended expanding the group counselling program for a larger HIV population.

G2N7: “It should continue and should address more people. You should make it accessible for those who are staying in rural areas.” (Female participant).

Counsellors also recommended expanding the intervention by involving other stakeholders.

C3: “Yes, it needs collaboration…because some of the problems require involvement of other stakeholders to support these people. Some participants may not have shelter, and some may not have food. Even some may need lawyers to solve their legal problems in courts. I would say most of our clients died not because of HIV but due to psychosocial and financial problems.” (Female counsellor).

### Depressive symptoms, QoL, functioning and perceived social support

Of 31 participants recruited at baseline, 28 of them were reassessed for depressive symptoms, perceived social support, QoL and functioning. As a parametric procedure, the data were fitted with paired samples *t* test assumptions. The descriptive statistics indicated that frequency, percentile, mean and standard deviation were normally distributed for each dependent variable when visualized in a histogram. The mean PHQ-9 depression scores reduced significantly between baseline and post-intervention (mean difference (MD) = 9.92, *t* =  − 7.82, *p* < 0.001). The mean perceived social support scores (MD = 0.79, *t* = 2.84, *p* = 0.009) and the mean QoL scores (MD = 0.39, *t* = 4.58, *p* < 0.001) improved significantly between baseline and post-intervention. However, there was no statistically significant difference in mean scores of functioning between baseline and post-intervention (MD = 1.27, *t* = 0.70, *p* = 0.492) (Table 6).

**Table 6:** Paired *t* test analysis: change in depressive symptoms, quality of life, functionality and social support among group IPT participants, FHRH, Northwest Ethiopia (*N* = 28)

Outcome	Baseline mean score	Post-group IPT mean score	Mean difference	*t* (95%CI)	*P* value	Effect size (Cohen’s *d*)
Depressive symptoms (PHQ-9)	16.77 (6.74)	6.74 (4.62)	− 9.92	− 7.82 (− 12.54, − 7.31)	< 0.001	1.74
Functionality (WHODAS-12)	36.81 (8.99)	38.08 (8.09)	1.27	0.70 (− 2.48, 5.02)	0.492	0.15
Perceived social support (PSS-12)	4.63 (1.36)	5.43 (0.92)	0.79	2.84 (0.22, 1.37)	0.009	0.69
Quality of life (WHOQOL-HIV-BREF-Eth)	3.23 (0.50)	3.67 (0.51)	0.39	4.58 (0.21, 0.56)	< 0.001	0.87

## Discussion

This study was the first to pilot the acceptability and feasibility of group IPT for treatment of depressive symptoms for PLWHA in Ethiopia and was perceived as being an acceptable and helpful intervention in addressing psychosocial problems and in reducing depressive symptoms. The procedures used during the intervention and the results of the intervention were highly acceptable as 93.5% (*n* = 29) of the participants completed the intervention. The context of the ART clinic with a highly stable workforce and the process of consultation on the need for and implementation of the intervention together with stakeholders, may have contributed to this positive outcome [36]. This positive finding may also be because of the use of peer counsellors, an accessible intervention setting and reimbursement of transportation costs. Transport costs were paid to compensate transport costs and the time spent to attend intervention sessions. Moreover, establishing a good therapeutic alliance during pre-group sessions can also have contributed to engaging the participants in the group sessions. This finding is supported by a randomized controlled trial from Uganda that demonstrated high acceptability and effectiveness of group IPT with an excellent acceptability (only 7.8% dropout rate) [37] and findings of other studies from LMICs [13, 38, 39]. The interpersonal theory that hypothesizes that IPT can decrease interpersonal stress and enhance social support through improving social skills, emotion processing and resolution of interpersonal problems [40] seems to hold promise for the HIV population.

The structure of the group IPT with a total of eight weekly group sessions was acceptable. However, some counsellors wished to increase the number of sessions to 10 or 12 to ensure that all participants improved from depressive symptoms believing that more depressive symptoms can be treated as the number of sessions are increased. Many previous studies have used up to 16 or more weekly sessions which could be important in producing long-term benefits [41]. The use of an extended number of sessions may be time consuming, labour intensive and resource demanding—and may also suffer from low acceptability. The high cost and low availability of trained personnel in LMICs encourages the use of adapted psychological interventions that are brief, structured and simple to be delivered by peers or lay counsellors rather than the complex interventions that demand more resources and time [42]. However, attention should be paid not to miss core elements such as the four IPT problem areas when adapting group IPT and other interventions [43].

The use of trained peer counsellors was acceptable as most of the group participants felt that peer counsellors can understand psychosocial problems of PLWHA and were happy with the skills and competency level of the counsellors. Both male and female counsellors were acceptable to facilitate group sessions either alone or together. However, they emphasized that supervision should be in place. This is supported by previous findings that peer counsellors successfully deliver psychological interventions [1, 20]. The randomized controlled trial from Uganda successfully used trained community health workers as counsellors who were also accepted by the community as they have common understanding and shared world views on depression [37].

Most of the participants were comfortable with the ART clinic setting, although one stated that she felt uncomfortable to come at the ART clinic due to “bad memories.” The ART clinic setting was probably in the most accessible location for residents in the town and it was chosen for a reason to maintain confidentiality and minimize stigma [21]. Therefore, the integration of mental health services such as group IPT within the services provided by the ART clinic seems an acceptable approach.

Generally, the group IPT was found to be feasible for use in larger HIV populations which is in agreement with reports of previous studies in LMICs [13, 37–39, 44]. However, it is important to note that several barriers could affect its implementation. Access to and cost for transportation, sickness, stigma, social factors and public employment were the main challenges that could affect similar interventions. Most of these barriers can be addressed if interventions are arranged based on the convenience of participants and if clients and counsellors show a strong commitment to the intervention. This includes deployment of adequate numbers of trained counsellors and high commitment of clients in minimizing social barriers.

From the quantitative results, we found a significant reduction of depressive symptoms, improvement in perceived social support and QoL at the post-intervention assessment. These findings need to be viewed with caution given the lack of a control group. Nevertheless, the quantitative findings were supported by in-depth interviews with group IPT participants—most of them said that they benefited greatly from the intervention. The significant reduction in depressive symptoms and improvement in QoL is supported by findings of several randomized controlled trials in LMICs [15, 37, 45], suggesting that increased social support mediates depressive symptoms that can lead to improved QoL. There was no significant improvement in functioning at the end of the intervention. The comorbidity of HIV/AIDS with depression could have a bigger effect than HIV/AIDS alone on functioning that may not be improved with group IPT alone.

IPT has been extensively examined and widely used for the treatment of depression in high-income countries. The basis for IPT is interpersonal theory which makes a practical link between mood disturbance and problematic life events [46]. In principle, IPT considers depression as a medical illness [46]; however, it does not specifically address biological risk factors. Thus, it has limited therapeutic scope for depression caused by biological factors, since its foundation is built on only interpersonal aspects of life. Despite its limitations, IPT has been found to be effective, acceptable and feasible as a therapeutic intervention, which is supported by rich evidence in other studies [47].

There are arguments among scholars on acceptable treatment approaches of IPT. Some scholars argue that a group-based approach is more acceptable and feasible than the individual-based approach [37, 48], while others favour individual-based intervention [49]. The findings in this study indicated that, despite its implementation barriers, group-based IPT can be an acceptable and feasible approach for PLWHA in Ethiopia. Common challenges for the implementation of group-based IPT in low-income settings may include (i) structural problems such as inconvenient intervention setting and (ii) unsupportive models of care such as health systems unfavourable to the integration of group-based interventions into the existing services. Implementation of group-based IPT could also be challenging when there is a lack of trust among the therapeutic team and if confidentiality becomes a concern for clients. The cost of the intervention could also be a challenge, but this needs further economic evaluation. However, cost-wise previous studies favour group-based intervention [48, 50].

Finally, the participants strongly recommended expanding the intervention in collaboration with other stakeholders such as with the government, non-governmental and charity organizations. Without collaboration, the intervention will remain inaccessible and limited to those who are located near the intervention setting and who can afford paying transport costs. Moreover, accessibility and affordability should be a priority when replicating psychological treatments including group interpersonal therapy.

### Limitations

Despite its important findings, our study had several limitations that should be considered while interpreting the results. First, our study had no control group to compare the results. Second, the sample included in the pilot intervention was small as the main aim of the study was to explore the acceptability and feasibility of the group IPT. A social desirability bias response from participants may have influenced the acceptability of the intervention. However, we used independent data collectors (research assistants) for the baseline and post-intervention interviews to minimize the social desirability bias. Third, our study does not show long-term effects of the intervention. Fourth, we have noted in the limitations section that scaling up of this intervention would need to consider including the costs of transport for participants.

## Conclusion

The intervention procedures and strategies of group IPT delivered by peer counsellors seemed acceptable and feasible for PLWHA in Ethiopia. PLWHA can benefit from a group IPT intervention in treating depressive symptoms in low-income settings. However, the training course for counsellors should include professional ethics to address gaps in attitudes and to capacitate counsellors in professionalism. We recommend that future studies focus on examining the effectiveness of peer-delivered group IPT for treatment of depressive symptoms using randomized controlled trials.

## Supplementary Information

**Additional file 1: Supplementary file 1.** Theory of Change on the implementation of group Interpersonal Therapy for depressed People with HIV/AIDS.

**Additional file 2: Figure.** Procedures used during the implementation of the intervention.

## Data Availability

Not applicable.
