# Supplementary material for: Acceptability and feasibility of peer-administered group interpersonal therapy for depression for people living with HIV/AIDS—a pilot study in Northwest Ethiopia
Source: Pilot Feasibility Stud. 2021 Jul 28;7:147. doi: 10.1186/s40814-021-00889-x (PMC8317371; doi:10.1186/s40814-021-00889-x)
Supplement: Supplementary file 1 — Additional file 1: Supplementary file 1. Theory of Change on the implementation of group Interpersonal Therapy for depressed People with HIV/AIDS. [file 40814_2021_889_MOESM1_ESM.docx]

**Supplementary file 1. Theory of Change on the implementation of group Interpersonal Therapy for depressed People with HIV/AIDS.**

| **Problems** | **Resources** | **Implementation** | **Outputs** | **Outcomes** | **Impact** |
| --- | --- | --- | --- | --- | --- |

Meeting space

Trained trainers

guidelines

audio-recorder

Motivated staff

Enabling culture

Depression

Non-adherence to ART

Poor QoL

Disability

Stigma

Poverty

Four IPT groups are formed

Screening PWHA using PHQ-9 and providing individual sessions for those who scored ≥5

Reduced disability (D)

Improved QoL (E)

**Assumptions**

1. Group IPT reduces depression
2. Treating depression improves social support
3. Treating depression improves adherence to ART
4. Treating depression improves functioning
5. Treating depression improves QoL
6. Increased CD4 leads to undetected viral load

20 to 40 participants are recruited

1 IPT Group includes 5 to 10 participants

32 group sessions are delivered

All group participants adhere to their ART treatment

Undetected viral load (F)

Increased adherence (C)

Increased social support (B)

Building trust among participants through keeping confidentiality

Supervision and fidelity check

Forming IPT groups

Recording sessions

Increased CD4

Reduced depressive symptoms (A)

Eligible PLWHA receive Group IPT
