# Supplementary material for: Acceptability and feasibility of peer-administered group interpersonal therapy for depression for people living with HIV/AIDS—a pilot study in Northwest Ethiopia
Source: Pilot Feasibility Stud. 2021 Jul 28;7:147. doi: 10.1186/s40814-021-00889-x (PMC8317371; doi:10.1186/s40814-021-00889-x)
Supplement: Supplementary file 2 — Additional file 2: Figure. Procedures used during the implementation of the intervention. [file 40814_2021_889_MOESM2_ESM.docx]

**Figure: Procedures used during the implementation of the intervention**
